# Tobacco Dependence Treatment in Oncology: Initial Patient Clinical Characteristics and Outcomes from Roswell Park Comprehensive Cancer Center

**DOI:** 10.3390/ijerph17113907

**Published:** 2020-05-31

**Authors:** Christine E. Sheffer, Jeffrey S. Stein, Cara Petrucci, Martin C. Mahoney, Shirley Johnson, Pamela Giesie, Ellen Carl, Laurie Krupski, Allison N. Tegge, Mary E. Reid, Warren K. Bickel, Andrew Hyland

**Affiliations:** 1 Roswell Park Comprehensive Cancer Center, Buffalo, NY 14263, USA; cara.petrucci@roswellpark.org (C.P.); martin.mahoney@roswellpark.org (M.C.M.); Shirley.Johnson@RoswellPark.org (S.J.); Pamela.Giesie@RoswellPark.org (P.G.); Ellen.Carl@RoswellPark.org (E.C.); Laurie.Krupski@RoswellPark.org (L.K.); mary.reid@roswellpark.org (M.E.R.); Andrew.Hyland@RoswellPark.org (A.H.); 2 Fralin Biomedical Research Institute at VTC, Roanoke, VA 24016, USA; jstein1@vtc.vt.edu (J.S.S.); ategge@vt.edu (A.N.T.); wkbickel@vtc.vt.edu (W.K.B.)

**Keywords:** cancer survivors, smoking, tobacco dependence, cognitive behavioral treatment

## Abstract

Despite the importance of smoking cessation to cancer care treatment, historically, few cancer centers have provided treatment for tobacco dependence. To address this gap, the National Cancer Institute (NCI) launched the Cancer Center Cessation Initiative (C3i). As part of this effort, this study examined implementation outcomes in a cohort of cancer survivors (CSs) who smoked cigarettes in the first year of an ongoing process to develop and implement a robust Tobacco Treatment Service at Roswell Park Comprehensive Cancer Center. We provide a comprehensive description of the new tobacco use assessment and referral process, and of the characteristics of cancer survivors who agreed to treatment including traditional tobacco-related psychosocial and cancer treatment-related characteristics and novel characteristics such as delay discounting rates. We also examine characteristic differences among those who agreed to treatment between those who attended and those who did not attend treatment. As the new tobacco assessment was implemented, the number of referrals increased dramatically. The mean number of treatment sessions attended was 4.45 (SD = 2.98) and the six-month point prevalence intention to treat abstinence rate among those who attended was 22.7%. However, only 6.4% agreed to treatment and 4% attended at least one treatment session. A large proportion of cancer survivors who agreed to treatment were women, of older age, of lower socioeconomic status (SES), and who had high levels of depressive symptomology. The findings demonstrate that the implementation of system changes can significantly improve the identification of cancer survivors who use tobacco and are referred to tobacco use treatment. Among those who attend, treatment is effective. However, the findings also suggest that a systematic assessment of barriers to engagement is needed and that cancer survivors may benefit from additional treatment tailoring. We present plans to address these implementation challenges. Systematic electronic medical record (EMR)-sourced referral to tobacco treatment is a powerful tool for reaching cancer survivors who smoke, but more research is needed to determine how to enhance engagement and tailor treatment processes.

## 1. Introduction

Despite immense progress in cancer survival rates, the number of new cancer diagnoses is steadily increasing, primarily due to advances in early detection and treatment as well as increased longevity in an aging population [1]. Within the next decade, over 20 million people in the U.S. will be cancer survivors (CSs), individuals who are undergoing or have completed cancer treatment, many of whom can achieve long-term benefits from healthy lifestyle changes [2,3,4,5,6]. Accordingly, the current and projected societal burden of cancer and its sequalae are staggering. Cancer care alone is estimated to cost $174 billion annually in 2020 [7]. Cancer treatment goals and models of survivorship care include strategies for optimizing cancer treatment outcomes by encouraging healthy behaviors [8]. 

Smoking cigarettes after any type of cancer diagnosis markedly decreases the efficacy of cancer treatment and the quality of life, increases the side effects of cancer treatment, and increases the risk of death from all causes [9,10,11]. Smoking following diagnosis adds to the cost of cancer care by increasing treatment modality failures, treatment complexity, and the probability of new or worsening comorbidities (e.g., cardiovascular disease, diabetes, osteoporosis) [12,13]. Smoking cessation, at any time after diagnosis, prolongs life and improves the quality of life for CSs [14,15,16,17]. Although the prevalence of smoking among CSs varies by cancer type [18,19,20], younger CSs are more likely to smoke than individuals without cancer [18,20]. The most recent estimates indicate that 29.2% of CSs aged 18–44 years smoke cigarettes, double the national prevalence of smoking in the U.S. [21]. 

Despite the importance of smoking cessation to cancer care treatment and the proliferation of robust smoking cessation treatment guidelines by the National Comprehensive Cancer Network (NCCN) [22], few of the premier comprehensive cancer centers in the USA have provided adequate treatment for tobacco dependence [23,24]. Only three out of sixty-two cancer centers reported system-wide tobacco treatment program outcomes in 2013 [23]; and only about half of CSs who smoke report having received any type of support for cessation in the past year [25]. To address the need to improve access to and the quality of tobacco treatment provided to CSs, the National Cancer Institute (NCI) launched the Cancer Center Cessation Initiative (C3i) as part of the Cancer Moonshot program [24]. Roswell Park Comprehensive Cancer Center received funding through this program in 2018 to improve the capacity and infrastructure to provide robust tobacco treatment services to all Roswell Park patients. 

At first glance, a life-threatening cancer diagnosis may appear to be a strong incentive to quit smoking; however, CSs face unique challenges to smoking cessation. CSs often incur significant physical, financial, and psychosocial burdens associated with cancer that strain coping and other resources [26,27]. Some CSs experience a sense of a foreshortened future and thoughts of early mortality, which reduce the perceived value of future planning [28,29,30]. A full understanding of the characteristics of CSs who seek treatment for smoking cessation has the potential to more fully inform the development of robust tobacco treatment programs tailored for CSs, assist NCI’s efforts to integrate the treatment of tobacco dependence as a standard of care for CSs [24], and perhaps identify therapeutic targets that are particularly pertinent to the treatment of tobacco dependence among CSs. 

This cohort study examined preliminary implementation and treatment outcomes from the first year of an ongoing process to develop a robust Tobacco Treatment Service at Roswell Park. Our goal is to develop a comprehensive Tobacco Treatment Service that includes systematic referral of all CSs who have used tobacco in the past 30 days and the proactive offer of integrated evidence-based treatment that addresses the unique psychosocial issues and concerns of CSs. To that end, we describe the impact of a new tobacco use assessment in the electronic medical record (EMR) on the volume of referrals; we provide a comprehensive description of the characteristics of CSs who agree to treatment; and we examine characteristic differences among those who agree between those who attend at least one session of treatment and those who do not. Understanding the characteristics of CSs can help address the unique psychosocial issues and concerns of CSs who use tobacco. Understanding characteristic differences associated with non-attending can help to identify potential barriers to treatment engagement and develop potential interventions to help CSs who use tobacco follow through with attending treatment. We include multiple demographic, tobacco use, clinical, and psychosocial characteristics known to be key to the treatment of tobacco use as well as novel characteristics such as delay discounting rates. End-of-treatment and six-month point prevalence-adapted intention to treat (ITT) and complete case analysis (CCA) abstinence outcomes are reported [31]. We then synthesize the findings in the context of other treatment cohorts and populations. Next steps and recommendations are then made to address challenges as we continue with implementation. 

## 2. Materials and Methods 

### 2.1. Study Design 

This study was approved by the Roswell Park Institutional Review Board. Data were extracted from a Windows-based expert tobacco treatment management software system developed and owned by Roswell Park called QuitClinic as well as the EMR Allscripts^®^ (Allscripts Healthcare, LLC, Chicago, IL, USA). Referral data included all referrals from 1 September 2018 to 30 November 2019. Treatment-related data included all CSs who completed a Tobacco Treatment Service intake assessment from 10 September 2018 to 2 October 2019. 

### 2.2. Source of Referrals to the Tobacco Treatment Service

Referrals are generated weekly from an EMR report of current tobacco users who visited an outpatient clinic in the past week. A slow roll-out of a revised tobacco use assessment in the EMR was implemented in the first quarter of 2019 with the goal of routine tobacco use assessment in the EMR conducted during all outpatient intake visits and repeated every 90 days. Changes in the process included streamlining the tobacco assessment, making the tobacco assessment a required element of the outpatient intake, and enabling CSs to complete the assessment on an iPad, along with other information, in the reception area prior to clinic visits. In the new system, all CSs who reported any tobacco use in the past 30 days were automatically referred via electronic transmission to the Tobacco Treatment Service. 

### 2.3. Tobacco Treatment Service

**Figure 1:**
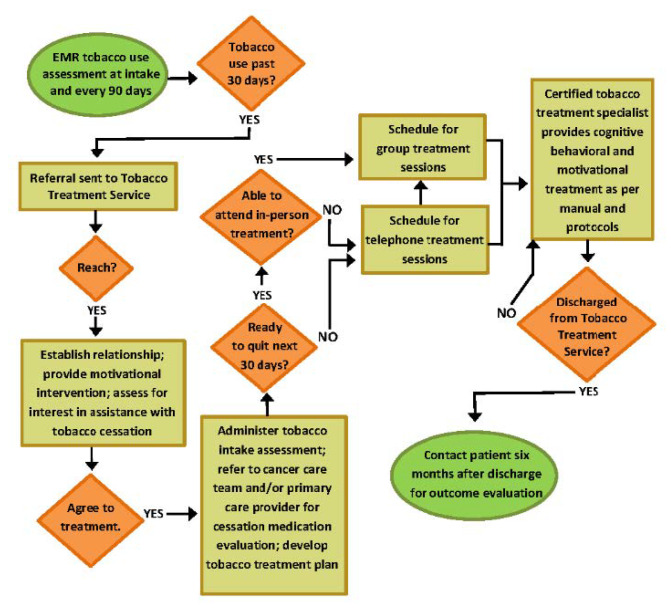
Tobacco Treatment Service workflow.

The Tobacco Treatment Service is staffed by 1.5 full-time certified Tobacco Treatment Specialists. Referrals and all patient interactions are managed via the QuitClinic expert system software platform. QuitClinic organizes referral lists, facilitates the administration of the treatment intake, tracks all patient contacts and interactions, and compiles and transmits patient notes from these interactions to the patient EMR. Referrals from the EMR Tobacco Use Assessment were received by the Tobacco Treatment Service weekly. All referrals were contacted within 1 week, with few exceptions. One attempt by telephone was made to contact all referrals within 1 week of the referral. CSs who agreed to treatment were administered the tobacco treatment intake assessment over the telephone, counseled to contact their cancer care provider and/or primary care provider for a cessation medication evaluation, and scheduled for their first tobacco treatment session. Treatment sessions are provided via telephone or via on-campus closed group in-person sessions. Group treatment was encouraged, but patients determined their ability to attend groups (see Figure 1).

### 2.4. Tobacco Treatment

The tobacco treatment intake assessment was administered prior to any tobacco treatment and included the assessment of multiple psychosocial characteristics that have been shown to be associated with treatment outcomes [32,33,34,35,36]. The intake results determined whether preliminary treatment sessions over the telephone were needed. The need to accommodate appropriate treatment for complex patients at all levels of readiness precludes a “one-size fits all” approach throughout treatment. Tobacco users must be ready to make a quit attempt in order to engage in a manual-driven, structured tobacco treatment approach. Preliminary treatment sessions prepared CSs to engage in the cognitive-behavioral and motivational treatment as per the treatment manual. For instance, CSs with high levels of psychological distress might receive a referral to social work and preliminary tobacco treatment sessions focused on how feelings of depression, anxiety, stress, or distress impact tobacco use. Similarly, CSs with low motivation levels might receive preliminary sessions focused on increasing and maintaining motivation; and CSs with high levels of nicotine dependence might receive preliminary sessions focused on pharmacotherapy-supported tobacco reduction strategies. These strategies were put in place to systematically tailor treatment, increase readiness to quit, and reduce the negative impact of psychosocial characteristics associated with poorer treatment outcomes.

When CSs were ready to make a quit attempt in the next 30 days, treatment sessions followed a well-established closed-session, weekly six-session, manual-driven, evidence-based, cognitive-behavioral approach [37]. CSs able to travel to campus were encouraged to attend group treatment. If unable to attend group treatment, the treatment manual was delivered via telephone. Cessation pharmacotherapy was addressed by referring CSs to their cancer care team and their primary care provider for a medication evaluation. If interested, CSs were also enrolled in the New York State Smokers’ Quitline to receive nicotine replacement and supplemental behavioral treatment. All CSs who were discharged from the Tobacco Treatment Service (e.g., completed treatment or no longer attending treatment sessions for any reason after engaging in treatment) were contacted for outcome assessment 6 months after they were discharged from the Tobacco Treatment Service (see Figure 1). 

### 2.5. Measures

The tobacco treatment intake assessment included standard demographic, tobacco use, and clinical measures. See Supplementary Materials for a detailed description of measures listed in Table 1. 

### 2.6. Outcome Assessment

Only CSs who received at least one treatment session were eligible for outcome assessment. Cigarettes per day was collected at each treatment contact. Cigarettes per day at the last treatment contact was used as the end-of-treatment outcome. Six-month outcomes were assessed by a specially trained interviewer by telephone six months after the end of treatment with the following items: “How many cigarettes are you smoking on a usual day?” and “Have you smoked a cigarette, even a puff, in the last 7 days?” 

### 2.7. Data Analysis

Referrals were quantified in terms of total number and number of unique referrals from September 2018 through November 2019 by month. The sample CSs who agreed to tobacco treatment were selected from referrals dated 10 September 2018 to 2 October 2019. CSs who agreed to treatment were characterized using descriptive statistics. Among those who agreed to treatment, characteristic differences between those who attended and did not attend treatment sessions were calculated using appropriate significance testing (analysis of variance (ANOVA) and χ^2^). Abstinence rates were calculated for end-of-treatment and 6-month 7-day point prevalence outcomes with 2 methods to accommodate missing data: (1) ITT, imputing all CSs lost to follow-up as smoking, and (2) CCA, eliminating participants lost to follow-up from the analysis [31,38]. Those who we were unable to contact due to death were eliminated from the denominator in the ITT abstinence ratio. 

## 3. Results

### 3.1. Referrals

**Figure 2:**
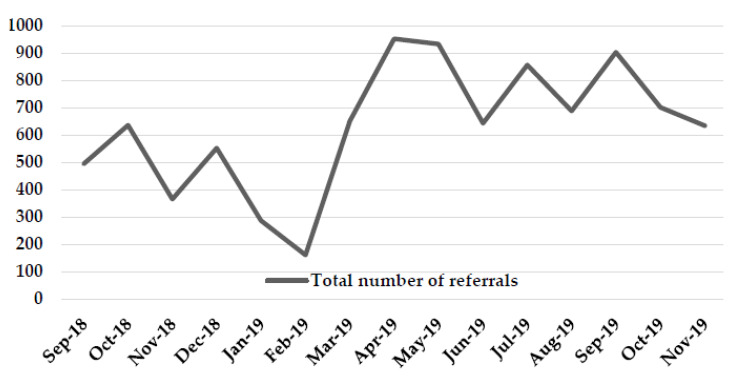
Roswell Park Tobacco Treatment Service Clinic Referral Volume.

From September 2018 to November 2019 (15 months), the Tobacco Treatment Service received a mean of *n* = 630 total referrals per month. In the first quarter of 2019, during the transition to the new tobacco use assessment, the number of referrals initially decreased but then increased dramatically. The mean number of monthly referrals from September 2018 to February 2019 was *n* = 416; the mean number of monthly referrals from March 2019 to November 2019 was *n* = 773 (see Figure 2).

### 3.2. Tobacco Treatment Engagement

Of the *n* = 8006 referrals received between 10 September 2018 and 2 October 2019, *n* = 5383 were unique patients, *n* = 344 (6.4%) agreed to treatment and were administered a tobacco treatment intake assessment, and *n* = 217 (4.0%) attended at least one treatment session. 

### 3.3. Participant Characteristics

Table 1 presents the characteristics of the *n* = 344 CSs who agreed to treatment and completed the tobacco treatment intake assessment, which includes *n* = 217 who attended at least one session of treatment and *n* = 127 who did not attend treatment. Additional details about the measures are provided in the Supplementary Materials. CSs were, on average, in late middle age; two thirds were women. The race and ethnicity of the CSs were generally reflective of western New York State. Most were of lower income but reported that their basic needs were mostly met. The mean time since initiating cancer treatment was 42.2 (SD 53.3) months with stage of cancer roughly equally distributed.

CSs showed moderate to high levels of nicotine dependence having smoked regularly for over 40 years with minimal periods of abstinence. Nearly 47% smoked menthol cigarettes. Although most had not attempted to quit recently, and nearly all intended to quit in the next 30 days. On average, they were highly motivated and moderately confident about their ability to quit. Only about one third had indoor home no-smoking policies. On average, CSs reported significant depressive symptomology, although stress levels were consistent with other smokers [39]. Perceived discrimination, a potential source of stress, was low overall, but a follow-up ANOVA found a significant difference in perceived discrimination between White and non-White CSs, (F 1343) = 20.9, *p* < 0.0001 (Whites mean = 1.1 (1.7) vs. Non-White = 2.2 (2.2)). Quality of sleep was within the normal range. Mean delay discounting rate was ln*k* = −4.7. Although the delay discounting rate for CSs with different cancer types appeared to vary (Supplementary Materials, Table S1), a follow-up ANOVA found no significant differences in discounting rates among cancer types, (F 10,310) = 0.77, *p* = 0.66. About 9% of CSs were in recovery from drugs and/or alcohol. The mean number of alcoholic drinks per week was 2.3 and CSs used cannabis, on average, three days in the past month. Two significant differences between those who attended treatment and those who did not were found—those who attended treatment had longer periods of abstinence in the past (20.41 months vs. 10.87 months) and were in the higher income categories ($35k–$74,999 and ≥$75,000). 

### 3.4. Preliminary Treatment Outcomes

Of the *n* = 217 who attended at least one treatment session and were discharged, the mean number of sessions attended was 4.25 (SD = 2.98; range 1–16; median = 3); *n* = 122 were at least six months post-discharge and eligible for the six-month outcome assessment. The six-month outcome assessment response rate was 46.7% (57/122). We were able to determine alive/death status data for 46% of CSs with intakes (158/344), *n* = 3 of whom were non-responders to the outcome assessment and eliminated from the ITT denominator. End-of-treatment cigarettes per day was available for all CSs, eliminating the need to impute smoking status at this milestone. At the end of treatment, 35.5% (77/217) of the CSs who received treatment were abstinent from smoking. Six months later, using CCA, 49.1% (28/57) were abstinent; using ITT analyses, 22.7% (27/119) were abstinent. 

## 4. Discussion

Our goal is to develop a comprehensive Tobacco Treatment Service at Roswell Park that includes systematic identification, referral, and treatment of all CSs who use tobacco and integrated evidence-based treatment that addresses the unique psychosocial issues and concerns of CSs. In the first year of implementation, the Tobacco Treatment Service increased the number of referrals dramatically; however, the numbers of CSs who agreed to treatment (*n* = 344) and those who engaged in treatment (*n* = 217) were small. Our findings provide meaningful insights into the implementation of effective processes for identification and referral of tobacco users as well as challenges with treatment engagement as we continue the ongoing process of developing and implementing a robust Tobacco Treatment Service at Roswell Park. 

The implementation of processes for the identification of CSs who use tobacco and the systematic referral to the Tobacco Treatment Service was a significant success as evidenced by the increase in the number of referrals. We attribute this success to a clear definition of tobacco use (e.g., use of any product in past 30 days), brief and face-valid assessment items in the EMR, a simple method of selecting one or more tobacco products, the ability of CSs to complete the assessment along with other routine questions on a clinic iPad in the waiting room, and making the tobacco use assessment mandatory for every CS every 90 days. Systematic identification and referral have been recommended as an evidence-based tobacco use treatment approach for over a decade [40] and automatic referral, in particular, has demonstrated remarkable effectiveness for connecting tobacco users with treatment resources [41]. However, the challenges with implementation in complex health settings with multiple competing priorities are many and the treatment of tobacco dependence remains neglected in cancer care [24]. Prior to the NCI Moonshot Cancer Center Cessation Initiate (C3i), fewer than half of the NCI-designated comprehensive cancer centers reported having systems to identify CSs who used tobacco [24]. Challenges included delineating tobacco treatment expectations and roles, justifying the importance of making the tobacco use assessment mandatory, harnessing information technology resources, and ensuring that the use of all tobacco products generate referrals. Our next steps will be to ensure the process is working as planned by examining the system and the data from different dimensions. 

Our findings reveal obvious challenges engaging CSs in treatment for tobacco dependence. Anticipating barriers, we provided treatment for CSs who are at any stage of readiness and developed flexible treatment approaches (see Figure 1) to accommodate low motivation, low-self-efficacy, depressed mood, high levels of nicotine dependence, and high levels of stress; and addressing barriers to access, we provided treatment free of charge and over the telephone and/or using in-person group treatment modalities. At present, the Tobacco Treatment Service makes one telephone contact attempt after a referral is received. Most attempts result in leaving a voicemail and most CSs do not return the call. Of those who agree to treatment, engage in an intake assessment, and are scheduled for treatment sessions, nearly all report that they are ready to make a quit attempt, but over a third do not ultimately attend any treatment sessions. The high level of readiness reported by CSs during the intake is offset by factors associated with significant challenges achieving abstinence that require therapeutic attention, including high dependence and depressive symptomology levels in addition to a failure to follow through with treatment. Follow-up analyses need to determine how relevant traditional assessments, including readiness to quit, are for CSs who are likely to feel pressured to quit smoking. Perceived readiness to quit may perhaps be confounded by perceived urgency and/or the need to respond favorably to providers and family. Patient characteristics suggest that we are able to engage CSs with a depressed mood, but we are not engaging CSs with lower levels of readiness, motivation, and self-efficacy. We are challenged to engage younger CSs (e.g., 18–44 years), who smoke at remarkably high prevalence rates [21], and men. Although there are few characteristic differences among those who agree and attend and those who do not attend, those who attend appear to have more experience with achieving abstinence in the past (20.41 vs. 10.87 months of abstinence), which may be indicative of higher levels of self-efficacy, and had fewer financial resources. Our next steps will be to examine the ramifications of these two known characteristics to engage CSs after they agree to treatment as well as examine other potential barriers to engagement throughout the process of tobacco use assessment to first treatment session. 

We plan to conduct a systematic assessment of barriers to treatment engagement complemented by a systematic examination of multiple strategies for increasing engagement. We plan to add a brief qualitative and quantitative assessment of barriers to the ongoing referral contact process as well as conduct a survey of potential barriers among all CSs referred in the past year. Proposed strategies for increasing engagement include the following: providing a brief message about the Tobacco Treatment Service after CSs complete the tobacco use assessment on the iPad; training nurses to support the referral during the patient interview in the exam room; contacting CSs by email, mail, and/or text in addition to telephone to offer tobacco treatment services; systematically providing CSs with a brochure or other materials before or after the first contact; revising the first contact language to be more persuasive or to focus on specific targets such as improving readiness to quit; providing reminder calls for the first treatment session; and offering treatment by video teleconference. Additional strategies will be proposed after the assessment of barriers is completed and analyzed. 

These findings suggest that many CSs who seek treatment for tobacco dependence have multiple characteristics that are associated with poorer outcomes, including lower socioeconomic status (SES), high levels of depressive symptomology, and female sex/gender [32,42,43]. The mean age was older than the mean age of smokers in the general population [44,45], of other treatment-seeking smokers [33], and of other treatment-seeking cancer CSs [46]. While older age is not a consistent predictor of tobacco treatment outcomes, older smokers often have a long smoking history, experience social isolation, are of lower income and education, and reside in deprived socioeconomic areas [47,48,49]. These characteristics may be important targets for tailoring approaches to increase treatment engagement and CS tobacco treatment-related needs. Treatment for women may highlight factors of importance to women including weight management concerns [50,51] and menstrual cycle phase [52], and sex/gender differences in medication choice. Varenicline is more effective and nicotine replacement (with the exception of the inhaler) is generally less effective for women than men [53,54]. Tobacco treatment may also add a standardized cognitive-behavioral mood management module for CSs with CES-D scores ≥ 16 [55]. 

Our findings suggest that delay discounting rates among CSs who agreed to treatment were lower than is typically reported for smokers, especially smokers of low SES [56,57,58]. Mean rates of delay discounting were ln*k* = −4.7 (untransformed *k* = 0.0091). Expressing this value as effective delay 50 (ED_50_), the delay at which a reward loses 50% of its value, $1000 lost half its value in a mean of 110 days [59]. Among smokers in the general population, the discounting rate is ln*k* = −4.0 to −2.9 and $1000 loses half of its value in only 18–50 days [60,61,62,63]. While lower discounting rates are, of course, indicative of improved treatment outcomes [35,36,64,65,66], the lower rates in this study may reflect a selection bias whereby CSs who discounted at higher rates were less likely to agree to attend treatment. If so, it would be of interest to ascertain whether intervening with delay discounting as a therapeutic target might be beneficial during the treatment engagement decision-making process. 

Other methods to sustainably increase the proportion of CSs who are exposed to an evidence-based tobacco treatment include repeatedly and broadly disseminating information about the Tobacco Treatment Service among providers and CSs; training all cancer care providers to support tobacco treatment engagement; and/or offering minimal treatment services, such as texting programs and self-help materials that provide treatment-related content and support and, importantly, continued contact with the Tobacco Treatment Service for assistance. Tobacco treatment among CSs may also benefit from coordinated efforts to reduce the stigma associated with tobacco-related cancers across the cancer care continuum [67].

We are especially challenged with developing a system that ensures that every CS is routinely offered cessation medication options and that this interaction is effectively tracked. Making this element more systematic, well-accepted, and routine will require provider education, enhanced clinical focus, and perhaps a patient visit focused on a cessation medication evaluation. Integration of a summary of the Tobacco Treatment Service intake information from QuitClinic into the EMR may also provide support for this important element of care. We are also challenged with the staffing capacity to contact all referrals within one week as well as the capacity to provide treatment to all CSs who engage in treatment. We are planning to increase this capacity by transferring the initial contacts to the hospital Patient Access scheduling department, which will require specialized training for Access interviewers. We are also planning to implement fee-for-service billing for tobacco treatment sessions, which will increase our capacity to increase staffing for the Tobacco Treatment Service. In addition, we are taking steps to ensure the ability to track cohorts of patients as they flow throughout each step in the workflow represented in Figure 1. Creative methods to identify, track, and integrate treatment-related data as patients are repeatedly offered tobacco treatment are needed. 

Abstinence outcomes confirm that intensive, evidence-based treatment for tobacco dependence is effective, similar to other studies that provide similar treatment [46], acknowledging the limitations given a lack of biochemical validation and moderate response rates. In this instance, the CCA appears inflated given that the end-of-treatment rate was 35.5%. Actual long-term abstinence rates are likely to lie between the ITT and the CCA abstinence rates, namely 22%–49%.

The development and refinement of the QuitClinic platform and its integration with the EMR are an important part of our ongoing success. The detailed tracking of referrals and treatment interactions provides unique opportunities to integrate intensive tobacco treatment in cancer care, conduct program evaluation, effectively examine significant changes in small planned tests of interventions, and contribute to the research. 

## 5. Conclusions

As the number of cancer survivors increases, the importance of optimizing cancer treatment outcomes by supporting the cessation of tobacco use becomes more salient. The findings from the first year of a new Tobacco Treatment Service at Roswell Park demonstrated that the implementation of system changes can significantly improve the number of tobacco users who are referred to treatment; however, more than a telephone call is needed to effectively engage CSs in tobacco treatment. These findings suggest that a systematic assessment of barriers to engagement is needed and that multiple characteristics are likely to be important targets for tailoring treatment engagement efforts and treatment approaches, including lower SES, high levels of depressive symptomology, female sex/gender, and older age. Systematic EMR-sourced referral to tobacco treatment is a powerful tool for reaching CSs who smoke, but more research is needed to determine how to refine and enhance engagement and treatment processes. 

## Figures and Tables

**Table 1:** Participant characteristics: Agreed to treatment (*n* = 344) including those who attended (*n* = 217) and those who did not attend (*n* = 127) treatment.

Variable	Category or Range	% (*N*) or Mean (SD)
Age, years	(21–83)	59.8 (10.2)
Sex	Female	68% (234)
Partnered status ^1^	Un-partnered	58.4% (201)
	Partnered	41.6% (143)
Race	White	73.8% (254)
	Black	18.3% (63)
	Multi-ethnic	3.5% (12)
	AI or AN ^2^	1.7% (6)
	Other	2.6% (9)
Ethnicity	Hispanic	4.4% (15)
Work status	Disabled	36.6% (126)
	Retired	33.1% (114)
	Full- or part-time	22.7% (78)
	Unemployed	5.5% (19)
	Homemaker	2.0% (7)
Household income *	≤$14,999	41.6% (143)
	$15,000–$34,999	29.4% (101)
	$35,000–$74,999	21.5% (74)
	≥$75,000	7.6% (26)
Education, years	(1–18)	12.5 (2.3)
Highest grade completed	Up to middle or junior high school (1–8)	2.3% (8)
	High school (9–12)	49.1% (169)
	College (13–16)	45.9% (158)
	Graduate school (17+)	2.6% (9)
Health insurance status	Medicare and/or Medicaid	72.7% (250)
	Private	27.0% (93)
	None	0.3% (1)
Basic needs met	(1–10)	8.6 (2.0)
Cancer health status		
Cancer stage	0	6.5% (8)
	I or II	53.2% (66)
	III or IV	40.3% (50)
ECOG ^3^	Grade 0	57.1% (188)
	Grade 1	36.5% (120)
	Grade 2	5.2% (17)
	Grade 3	0.9% (3)
	Grade 4	0.3% (1)
Cancer treatment	Surgery	65.0% (102)
	Chemotherapy	56.4% (88)
	Radiation	21.0% (33)
	Hormone therapy	24.4% (38)
	Immunotherapy	10.3% (16)
Tobacco use measures		
CPD ^4^	(0–50)	15.7 (8.8)
Smokeless tobacco	Yes	2.6% (9)
FTND ^5^	(0–10)	4.7 (2.1)
Menthol use	Yes	46.6% (160)
Age started	(6–56)	16.6 (5.4)
Years smoking	(1–70)	40.2 (12.1)
Longest period of abstinence, months *	(0–252)	16.9 (34.2)
Plans to quit smoking cigarettes	Already quit	0.6% (2)
	Quit cigarettes, but use other tobacco products	1.2% (4)
	Within 30 days	97.4% (334)
	Within 6 months	0.3% (1)
	Beyond 6 months	0.3% (1)
	No plans	0.3% (1)
Last attempt to quit cigarettes	More than 12 months ago	59.9% (205)
	6 to 12 months ago	15.5% (53)
	3 to 6 months ago	5.6% (19)
	1 to 3 months ago	8.2% (28)
	Within the last month	5.3% (18)
	Never	5.6% (20)
Motivation ^6^	(0–10)	8.3 (2.1)
Self-efficacy ^6^	(0–10)	6.6 (2.5)
Weight gain concern ^6^	(0–10)	3.2 (3.9)
Smoking policy at home	No smoking in the home	37.4% (128)
Prior professional help quitting?	Yes	31.4% (107)
Clinical measures		
CES-D ^7^	(6–51)	17.6 (8.2)
PSS-4 ^8^	(0–16)	5.0 (3.9)
ESS ^9^	(0–24)	5.6 (4.2)
MSPSS ^10^	(20–70)	57.6 (11.2)
EDS ^11^	(0–9)	1.4 (1.9)
Delay discounting (log *k*)	(−9.1 to 3.2)	−4.7 (2.3)
In recovery	Yes	9.1% (31)
Drinks in one sitting	(0–10)	1.2 (1.8)
Drinks per week	(0–70)	2.3 (6.5)
Cannabis use, days per month	(0–30)	3.1 (8.2)
Physical activity	Yes, past 30 days	89.2% (297)
Min per activity	(0–500)	55.6 (68.0)
Activities per month	(0–120)	23.9 (14.0)

^1^ Un-partnered = single, divorced, separated, widowed; Partnered = married or living with significant other; ^2^ AI = American Indian, AN = Alaskan Native; ^3^ The Eastern Cooperative Oncology Group Performance Status; ^4 ^ CPD = Cigarettes per day; ^5^ Fagerstrom Test for Nicotine Dependence; ^6^ Assessed on a 0–10 scale with 0 = “not at all” and 10 = “most ever”; ^7^ Center for Epidemiologic Studies Depression Scale; ^8^ Perceived Stress Scale 4; ^9^ Epworth Sleepiness Scale; ^10^ Multidimensional Scale of Perceived Social Support; ^11^ Everyday Discrimination Scale. * Differences between those who attended treatment and those who did not, *p* ≤ 0.05. See Supplementary Materials for details regarding the measures.

## Supplementary Materials

The following are available online at https://www.mdpi.com/1660-4601/17/11/3907/s1, Tobacco Dependence Treatment in Oncology: Detailed Description of Measures, Table S1: Delay discounting of cancer survivors treated for tobacco dependence by cancer type (*n* = 311).
