# Supplementary material for: Tobacco Dependence Treatment in Oncology: Initial Patient Clinical Characteristics and Outcomes from Roswell Park Comprehensive Cancer Center
Source: Int J Environ Res Public Health. 2020 May 31;17(11):3907. doi: 10.3390/ijerph17113907 (PMC7312979; doi:10.3390/ijerph17113907)
Supplement: Supplementary file 1 [file ijerph-17-03907-s001.pdf]

International Journal of
Environmental Research and Public Health

MDPI

Supplementary Materials

Tobacco Dependence Treatment in Oncology: Detailed Description of Measures

Demographic measures included age, partnered status, race, ethnicity, education, income, employment status, and perceived deprivation of basic needs assessed with the question, "In the last 6 months, to what degree did you feel that your basic needs such as healthy food and safe housing are being met?" Health care insurance status was grouped into Medicare and/or Medicaid, Private, or None.

Cancer health measures were extracted from the cancer health registry and included cancer staging group (I-IV), the Eastern Cooperative Oncology Group Performance Status (ECOG), and type of cancer treatments received (surgery, chemotherapy, radiation, hormone therapy, and immunotherapy). Cancer staging groups were defined as follows: 0 = abnormal cells are present only in the layer of cells in which they have developed; I = cancer is small and not grown into nearby tissues or lymph nodes; II and III = progressively larger cancer that has grown into nearby tissues or lymph nodes; IV = cancer has spread to other parts of your body. The ECOG Performance grades were defined as follows: 0 = fully active, able to carry on all pre-disease performance without restriction; 1 = restricted in physically strenuous activity but ambulatory and able to carry out work of a light or sedentary nature; 2 = ambulatory and capable of all self-care but unable to carry out any work activities, up and about more than 50% of waking hours; 3 = capable of only limited self-care, confined to bed or chair more than 50% of waking hours, and 4 = completely disabled, cannot carry on any self-care, totally confined to bed or chair [1]. Time since diagnosis was calculated by subtracting date of first active treatment from the intake date.

Tobacco use measures included cigarettes per day, use of smokeless tobacco and mentholated cigarettes, duration/timing of last quit attempt, readiness to quit, age began smoking, years of regular smoking, and smoking policy in the home. Smoking policies were assessed with the following: (a) no smoking anywhere inside or outside, (b) no smoking inside, but smoking is allowed outside, (c) smoking is allowed in certain areas inside, or (d) smoking allowed anywhere inside [2]. Motivation to quit, self-efficacy for quitting, and concern about weight gain after quitting were assessed on a 0–10 scale with 0 = "not at all" and 10 = "most ever" and the following questions: "How much do you want to quit smoking?", "How confident are you that you can quit using tobacco and stay quit for good?", and "How concerned are you about gaining weight after you quit?"[3–6]. Nicotine dependence was assessed with the 6-item Fagerström Test for Nicotine Dependence, range 0–10. Higher scores indicate greater dependence levels and a lesser likelihood of achieving abstinence [7].

Clinical measures were included because of their relevance to tobacco treatment. The Perceived Stress Scale 4 (PSS-4) assessed stress in the past 30 days with 4 items using a 5-point scale where 0 = "never" and 4 = "very often", range 0–16. Mean levels for smokers ranged from 4.8 to 5.9 [8]. The Centers for Epidemiological Studies Depression Scale (CES-D) assessed past week distress and depressive symptomatology with 20 items on a 4-point scale where 0 = "rarely or none of the time" and 3 = "most or all of the time", range 0–60. Items assess depressed mood, feelings of guilt and worthlessness, feelings of helplessness and hopelessness, psychomotor retardation, loss of appetite, and sleep disturbance. A score of 16 or greater indicates possible depression [9]. The William's Everyday Discrimination Scale (EDS) assessed discriminatory experiences with 9 items on a 4-point scale where 0 = "never" and 3 = "often", range 0–27. Higher scores indicate more exposure to discrimination and are associated with poorer physical health [10]. Sleep is often affected by stress. The Epworth Sleepiness Scale (ESS) assessed daytime sleepiness, a strong indicator of sleep quality, with 8 items on a 4-point scale where 0 = "never" and 3 = "high chance of dozing", range 0–24. Higher scores indicate a higher likelihood of poor nighttime sleep; 0–5 = Lower Normal Daytime Sleepiness; 6–10 Higher Normal Daytime Sleepiness; 11 or 12 Mild Excessive Daytime Sleepiness; 13–15

Moderate Excessive Daytime Sleepiness; 16–24 Severe Excessive Daytime Sleepiness [11]. Physical activity was assessed with three items from the Behavioral Risk Factor Surveillance System (BRFSS) assessing the frequency and number of minutes of physical activity in the past month. Greater physical activity is associated with better quality of sleep, improved mood, and improved judgment skills [12]. The Multidimensional Scale of Perceived Social Support (MSPSS) is a 12-item measure comprised of three subscales assessing levels of perceived social support from family, friends, and significant other on a 7-point scale where 1 = "very strongly disagree" and 7 = "very strongly agree". [13,14]. Greater values indicate greater levels of social support. Substance use history was assessed by asking whether patients considered themselves in recovery from drugs or alcohol. Alcohol use was assessed in number of drinks in a week and in one sitting. Cannabis use was assessed in days of cannabis use in the past month.

Delay discounting of monetary rewards has shown robust generality and predictive validity for smoking, level of dependence, and tobacco treatment outcomes in a variety of smoking populations [15–18], and is, in fact, a promising new therapeutic target for smoking cessation. Smokers with and without cancer significantly discount the value of monetary and health rewards more than non-smokers [19,20]. At present, however, the relations among delay discounting and other characteristics of CSs who smoke is virtually unexplored. Delay discounting rate was assessed with the 5-trial Adjusting Delay Discounting task, $1,000 magnitude, an interactive instrument that automatically adjusts to respondents' choices to produce a *k*-parameter after five trials [21]. Respondents were asked on the first trial whether they would prefer $500 now or $1000 in three weeks. If the immediate option was selected, then the second trial shortened the delay to one day ($500 now or $1000 in one day). If the delayed option was selected on the first trial, then the second trial lengthens the delay (i.e., $500 now or $1000 in two years). Delays on all subsequent trials are adjusted based on responses from the preceding trial. The *k*-parameter was expressed as the natural logarithm of *k* in Mazur's (1987) hyperbolic discounting model, with *k* increasing as the preference for smaller sooner rewards increases [22].

Table S1. Delay discounting of cancer survivors treated for tobacco dependence by cancer type (n = 311).

Primary cancer site	% (n)	ln$k$ (SD)
Head and Neck	12.2% (35)	−4.9 (2.1)
Lung and Esophagus	12.2% (39)	−4.8 (1.9)
Genitourinary	11.9% (39)	−4.7 (2.7)
Other Thoracic	11.9% (37)	−4.4 (2.3)
Breast	10.3% (34)	−4.3 (2.6)
Gynecologic	10.3% (33)	−4.5 (3.0)
Gastrointestinal	9.4% (30)	−4.3 (1.9)
Skin	7.3% (24)	−5.7 (2.5)
Blood Cancers	7.0% (22)	−4.8 (1.9)
Brain	5.2% (15)	−4.4 (2.5)
Soft Tissue	2.4% (8)	−4.7 (2.1)

References for Supplementary Materials

1. Oken, M.M., et al., Toxicity and response criteria of the Eastern Cooperative Oncology Group. *Am. J. Clin. Oncol.* **1982**, *5(6)*, 649–655.
2. Messer, K., et al., The effect of smoke-free homes on smoking behavior in the U.S. *Am. J. Prev. Med.* **2008,** ***35****(3)*, 210–216.
3. Perkins, K.A., Smoking cessation in women. Special considerations. *CNS Drugs* 2001. **15**(5), 391–411.
4. Moolchan, E.T., et al., Characteristics of African American teenage smokers who request cessation treatment: Implications for addressing health disparities. *Arch. Pediatrics Adolesc. Med.* **2003**, **157**(6), 533–538.
5. McKee, S.A., et al., Perceived risks and benefits of smoking cessation: Gender-specific predictors of motivation and treatment outcome. *Addict. Behav.* **2005**, **30**(3), 423–435.

6. McCarthy, D.E., et al., A multi-level analysis of non-significant counseling effects in a randomized smoking cessation trial. *Addiction* **2010**, **105**(12), 2195–2208.
7. Fagerstrom, K.O., et al., Nicotine dependence versus smoking prevalence: Comparisons among countries and categories of smokers. *Tob. Control* **1996**, **5**(1), 52–56.
8. Cohen, S. and E. Lichtenstein. Perceived stress, quitting smoking, and smoking relapse. *Health psychology* **1990**, **9**(4), 466–478.
9. Radloff, L.S., The CES-D Scale: A Self-Report Depression Scale for Research in the General Population. *Appl. Psychol. Meas.* **1977**, **1**(3), 385–401.
10. Williams, D.R., et al., Racial Differences in Physical and Mental Health: Socio-economic Status, Stress and Discrimination. *J. Health Psychol.* **1997, 2**(3), 335–351.
11. Johns, M.W., A new method for measuring daytime sleepiness: The Epworth Sleepiness Scale. *Sleep* **1991**, **14**(6), 540–545.
12. Harris, C., et al., Adult participation in aerobic and muscle-strengthening physical activities—United States, 2011. *MMWR Morb. Mortal. Wkly. Rep.* **2013, 62**(17), 326–330.
13. Zimet, G.D., et al., The mutidimensional scale of perceived social support. *J. Personal. Assess.* **1988**, **52**(1), 30–41.
14. Dahlem, N.W., G.D. Zimet, and R.R. Walker, The Multidimensional Scale of Perceived Social Support: A confirmation study. *J. Clin. Psychol.* **1991**, **47**(6), 756–761.
15. Sheffer, C.E., et al., Delay discounting rates: A strong prognostic indicator of smoking relapse. *Addict. Behav.* **2014**, **39**(11), 1682–1689.
16. Sheffer, C.E., et al., Delay discounting, locus of control, and cognitive impulsiveness independently predict tobacco dependence treatment outcomes in a highly dependent, lower socioeconomic group of smokers. *Am. J. Addict.* **2012**, **21**(3), 221–232.
17. Barlow, P., et al., Time-discounting and tobacco smoking: A systematic review and network analysis. *Int J Epidemiol* **2017,** *46*, 860–869.
18. Odum, A.L., Delay discounting: I'm a k, you're a k. *J. Exp. Anal. Behav.* **2011**, **96**(3), 427–439.
19. Sheffer, C.E., et al., The treasure of now and an uncertain future: Delay discounting and health behaviors among cancer survivors. *Cancer* **2018**, **124**(24), 4711–4719.
20. Baker, F., M.W. Johnson, and W.K. Bickel, Delay discounting in current and never-before cigarette smokers: Similarities and differences across commodity, sign, and magnitude. *J. Abnorm. Psychol.* **2003**, **112**(3), 382–392.
21. Koffarnus, M.N. and W.K. Bickel, A 5-trial adjusting delay discounting task: Accurate discount rates in less than one minute. *Exp. Clin. Psychopharmacol.* **2014**, **22**(3), 222–228.
22. Mazur, J.E., An adjusting procedure for studying delayed reinforcement, in The Effect of Delay and of Intervening Events on Reinforcement Value, Quantitative Analyses of Behavior, J.E.M. M.L. Commons, J.A. Nevin, H. Rachlin, Editor. 1987, Erlbaum: Hillsdale, NJ, USA; p. 55–73.
